# The influence of training experiences on career intentions of the future GP workforce: a qualitative study of new GPs in England

**DOI:** 10.3399/bjgp19X703877

**Published:** 2019-05-21

**Authors:** Sharon Spooner, Louise Laverty, Kath Checkland

**Affiliations:** Centre for Primary Care Research, School of Health Sciences, University of Manchester, Manchester.; Centre for Primary Care Research, School of Health Sciences, University of Manchester, Manchester.; Centre for Primary Care Research, School of Health Sciences, University of Manchester, Manchester.

**Keywords:** careers, general practice, medical education, qualitative research, recruitment and retention, specialty training

## Abstract

**Background:**

The capacity of the UK GP workforce has not kept pace with increasing primary care workloads. Although many doctors successfully complete GP specialty training programmes, some do not progress to work in NHS general practice.

**Aim:**

This article explores the training experiences and perceptions of newly qualified GPs to understand how their education, training, and early experiences of work influence their career plans.

**Design and setting:**

A qualitative study of doctors in their final year of GP training (ST3) and within 5 years of completion of GP training (F5).

**Method:**

Participants across England were recruited through training programmes, First5 groups, and publicity using social media and networks. Open narrative interviews were conducted with individuals and focus groups. Audiorecorded interviews were transcribed, and a thematic analysis was supported by NVivo and situational analysis mapping techniques.

**Results:**

Fifteen participants engaged in individual interviews and 10 focus groups were carried out with a total of 63 participants. Most doctors reported that training programmes had prepared them to deal confidently with most aspects of routine clinical GP work. However, they felt underprepared for the additional roles of running a practice and in their understanding of wider NHS organisational structures. Doctors wished to avoid unacceptably heavy workloads and voiced concerns about the longer-term sustainability of general practice.

**Conclusion:**

Strategies to attract and retain enough GPs to support delivery of comprehensive primary care should consider how doctors’ early career experiences influence their career intentions. A coherent plan is needed to improve their preparation and increase confidence that they can achieve a professionally satisfying, effective, and sustainable career in NHS general practice.

## INTRODUCTION

In the UK, it has become increasingly difficult for primary care providers to recruit sufficient GPs to keep pace with the growing demands of rising workloads.^1^^,^^2^ In the context of a global shortfall of trained family doctors,^3^^,^^4^ initiatives to improve recruitment have not been effective.^5^ Instead, workload pressures and low morale have been accompanied by an increase in early retirement rates and increased concerns about the sustainability of current models of primary care.^2^^,^^6^^–^9

The future UK GP workforce is significantly dependent on the recruitment and retention of approximately 3000 doctors who complete GP specialty training each year. This article discusses how insights from the training experiences of doctors emerging as new GPs could inform improvements in their training experiences and enhance successful transition from training to a full range of GP roles.

### Training requirements and objectives

Like other organisations that use training and development to achieve a workforce suited to their present and future needs,^10^ GP training programmes aim to produce new GPs ready to join the GP workforce.^11^ In addition, new GPs are officially required to engage with continuing professional development to adjust their professional practice in response to innovations in medical treatments and service models and modes of delivery.^12^

In the UK, doctors who have obtained a medical degree (4–5 years) undertake a 2-year foundation programme of medical work under supervision. Those wishing to become GPs undertake a 3-year (minimum) GP specialty training programme. This is guided by a core curriculum, which defines learning outcomes and competences relevant for entering independent NHS GP practice and supporting GPs’ future professional development.^11^ Doctors who are successful in all three components of the Membership examination of the Royal College of General Practitioners (RCGP) (Applied Knowledge Test, Clinical Skills Assessment, and Workplace-Based Assessment), and who meet the requirements for a Certificate of Completion of Training (CCT) are eligible for independent GP work.

Clinical training takes place in approved posts in hospital, general practice, or other community settings. GP specialty trainees record evidence of their learning and reflective analysis of their professional development using an ePortfolio, which is reviewed regularly to monitor progress towards the required competences of the training programme. Once qualified, their engagement with continued professional development is monitored via an ongoing portfolio and annual appraisals, with recertification to practice (revalidation) every 5 years.

**How this fits in**

Training programmes for GPs have remained substantively unaltered during a period of rapid change in how general practice is organised and resourced in the UK. In the context of recruitment and retention difficulties, better understanding of the preparation of emerging GPs and their career preferences is of vital importance. This article reports on wider aspects of training and other factors that influence career planning for recently trained GPs in England.

### Potential roles for GPs

Since the formation of the NHS, GPs have been involved in non-clinical roles as independent contractors delivering NHS-funded primary care services to groups of registered patients. Under the traditional GP partnership model, arrangements for premises, staffing, and funding are managed by GPs as business partners. However, since the early 2000s, the introduction of alternative service models, as well as a new standard General Medical Services contract, has supported a diversification in practice contracts and approaches, including, for example, very large multisite practices, and a significant increase in salaried practice. As the proportion of GPs holding partnership positions has reduced, the viability of continuing with a service model based around being an independent contractor has been debated,^13^ and a government review has recently been commissioned.^14^

In addition to well-established involvement in medical education, policy-driven changes in primary care have necessitated further diversification of roles for GPs; prominent examples include involvement in annual appraisal processes, and in healthcare commissioning and provider organisations.^15^^,^^16^

In these changing contexts, this article considers how new GPs reflect on their training programmes as preparation for the broad range of the roles and responsibilities undertaken by GPs and on how training experiences affect their continuing motivation and career intentions.

## METHOD

A qualitative approach was adopted to explore this topic in depth. Doctors were recruited in the final year of a GP training programme (ST3) or within 5 years of obtaining CCT (F5) as participants in individual and focus group interviews. Recruitment methods included invitations relayed by training programme organisers, F5 groups, open invitations shared via social media, and *ad hoc* contacts. Participants from a range of ethnic backgrounds and with a sex balance similar to UK GP training programmes^17^ were recruited across seven English regions.

An open, narrative style of interviewing approach was used to encourage new GPs to reflect broadly on their experiences of training in general and how these have shaped their career plans after obtaining CCT. A topic guide was used to prompt breadth and depth, and was iteratively developed during fieldwork. Interviews were audiorecorded and transcribed, and a thematic analysis was supported by NVivo (version 11) and situational analysis mapping techniques.^18^ Extracts from interview transcripts are labelled according to the participant’s career stage, individual or focus group ID, and sex (for individual interviewees only) (Box 1).

**Box 1.** Examples illustrating the meaning of participant ID coding pattern

**Participant code**	**Meaning of participant coding pattern**		
	**Career stage**	**Interview ID**	**Sex**
ST3F.F	3rd year GP specialty training	Individual F	F
F5A.M	Within first 5 years after CCT	Individual A	M
ST3 FG6	3rd year GP specialty training	Focus Group 6	–
F5 FG3	Within first 5 years after CCT	Focus Group 3	–

*CCT* = *Certificate of Completion of Training.*

The focus of results presented in this article is on those aspects of GP training that new GPs perceive as being important or as having a direct impact on their level of preparation for and motivation to become part of the future GP workforce.

## RESULTS

A summary of the participants’ information is shown in Table 1.

**Table 1.** Numbers of interview participants

**Type of GP**	**Individual interviews, *n***	**Focus groups, *n* (participants)**	**Ratio male:female, *n***
ST3	6	7 (50)	2:4
F5	9	3 (13)	4:5
Total	15	10 (63)	21:42
		Total sample = 78	27:51

### Preparation for clinical GP work

Interviews with new GPs indicated that their main focus was on learning how to be GPs. However, many reported that working in hospital-based specialties did little to prepare them for GP work. Many described being excluded from training opportunities provided for specialty trainees working alongside them in hospitals, and perceived hospital posts as mostly ‘service provision’ with limited useful learning opportunities. Describing their essential roles as ‘triage monkeys’ or ‘rota pluggers’, they described feelings of being unrecognised and undervalued:

‘I learnt more about being a GP in my GP rotations than I did in the hospital. In the hospital … I wasn’t necessarily absorbing what would be beneficial for me or my patients in primary care.’

(F5I.F)

‘I just wish the secondary care stuff was more … could be more tailored to GP, to actually what we’re going to be doing rather than just because we are very much rota pluggers as GP trainees and that hospital would absolutely keel over if it didn’t have GP trainees.’

(ST3F.F)

‘In terms of the things that I felt that were most useful, the outpatient-based rotations, like outpatient psychiatry I thought was very useful, those type of things really.’

(F5K.M)

In contrast, general practice training posts were seen as ‘world-class training’, built around one-to-one supervision by a GP trainer; regular, self-directed teaching; guidance before exams; and close clinical support:

‘I think the GP jobs are brilliant because … I have some sessions where I videotape my surgeries to help me get assessments, I have some sessions that are shared where myself and one of the other GPs … learn who would do what differently and why.’

(ST3F.F)

‘We’d have had a one-to-one … time together, every week, where she would just teach me stuff basically, in a tutorial fashion.’

(F5H.M)

Hospital duties often prevented doctors from attending GP-focused weekly training events where discussion of selected topics could close learning gaps but doctors could feel short-changed by an overemphasis on self-directed learning:

‘They’re good at picking the stuff that we struggle with and because they know where our curriculum gaps tend to be … like genetics and community orientation are quite hard to get sometimes.’

(ST3F.F)

‘In other training programmes, you’ve got your training day, you get the consultants coming in and teaching you, whereas here, we have mostly ST1s and us teaching ourselves. So whether we have GPs with a special interest coming and teaching us, which can be quite inspiring as well.’

(ST3 FG1)

Unlike hospital consultants, GP trainers were invested in supporting GP specialty trainees who may become future colleagues, providing high-quality, safe, comprehensive, and responsive care for patients in general practice:

‘… we want to make you better and because we’re holistic as a GP, we look at everything and we want to mend everything, and we’re taking on all that social problem, financial problems and, you know, social care. And I don’t think there’s a way you could be a good GP and not do that.’

(F5 FG1)

In general practice, doctors appreciated being released from the restrictions associated with hospital posts such as shift work, inflexible rotas, and moving from one place to another, but some felt isolated, and on occasion not adequately supported:

‘People have very varying experiences and some of us just were given a list to do … and sometimes the trainer wasn’t there at all, so we were unsupervised.’

(F5L.F)

‘My colleagues were still doing hospital jobs … and they still had this really good sort of camaraderie in work, whereas I was in a GP practice where the GPs were all a lot older than me.’

(ST3J.F)

Doctors felt that completion of their ePortfolio record of training was onerous and found limited benefit in recording lengthy reflections that were never discussed. They also reported a great deal of pressure from preparing for the professional examinations that were essential for successful completion of training (that is, Membership of the RCGP):

‘I was just like I don’t know enough … and I need to learn more but I don’t have time … I’m doing this stupid portfolio and I’ve got a life to live and poorly parents, and this, that, and the other.’

(F5C.M)

### Preparation for non-clinical GP roles

Several participants spoke of the challenges of ‘stepping up’ for work after obtaining CCT. Many felt that gaining clinical confidence took priority and were reluctant to consider the unknowns of partnership roles:

‘When I’m first qualified I’m aware that it’s going to be a bit of a step up to go from this nice, protected GP registrar role to a salaried … I don’t want to be trying to learn how to run a practice at the same time as trying to learn how to be a good GP.’

(ST3F.F)

‘I would like to be salaried and just get to grips with mastering being a GP before I think about anything else.’

(ST3 FG6)

Opportunities to observe and understand practice business varied widely; only those included in GP partners’ business meetings discovered how business matters, strategies, and priorities were discussed:

‘They let all the registrars get involved with all of their meetings … knowing how they run their practice and their business.’

(ST3 FG7)

*‘I thought I would be more involved in the management and I thought they would train me to be involving* [sic] *in managing the practice, both financially, organisationally, whereas that was a very closed shop so I didn’t ever get into any of the management meetings.’*

(F5A.M)

Participants expressed concern that recognised that they were insufficiently prepared for the eventual transfer of partnership responsibilities and unable to do so without incalculable risks:

‘We are the next generation who are going to be taking over at some point, aren’t we … so we need to know how things are done.’

(F5I.F)

‘We’re learning to be GPs, we’re not learning to be business people and … it’s nearly a million pounds’ worth of money that’s coming in and out of your practice and you’ve got lots of staff to employ and there are lots of responsibilities and … the payment structure for general practice is so complicated in terms of all your different payment streams into the practice. I think it’s those bits that scare me.’

(ST3F.F)

Many new GPs therefore chose salaried GP positions or locum employment where duties and responsibilities were generally clinically oriented and familiar.

Doctors felt ill informed about external bodies such as clinical commissioning groups (CCGs) and federations but some who had attempted to find out more found meetings unhelpful or troubling:

‘I went to a federation meeting, which was good to see but I felt like a lot of it was over my head, so definitely agree that we’re not taught enough to be able to step into that role.’

(ST3 FG6)

‘Last Thursday we had our protected learning time for the CCG. It was awful. It was terrible. And most of it was about how can we save this £9 billion we’ve got to save before April, that we get every single month.’

(F5 FG1)

They were aware that only a limited number of GPs were interested in such roles and experienced a tension about spending time in meetings rather than carrying out clinical work.

### Motivation to work as an NHS GP

A wide range of factors influencing new GPs’ immediate and longer-term career plans emerged in this study. These can be broadly categorised as wider organisational/structural elements, workload issues, and individual preferences.

#### Wider organisational/structural influences

During their medical training, participants have been aware of significant policy-driven shifts that impact on general practice. These have generated uncertainty and suspicion about how GP work may alter as their careers progress and made some, who would otherwise like to become partners, reluctant to make such a commitment:

‘It’s quite scary. It has actually made me think twice about being a partner. But because I like running the practice rather than seeing patients, I don’t mind being the one that doesn’t see the patients and does all the running it. So I’m going to do it probably. But, yeah, knowing the political climate, I don’t even know how to go about knowing the political climate.’

(F5 FG1)

‘I don’t see how a primary care structure that runs on multiple small businesses is gonna be cost-effective to continue. So I don’t see why I should put my own money into running something, when it could all change in the next 5 years.’

(ST3 FG2)

More fundamentally, doctors expressed concerns about whether the values and ethos they associate with NHS care would continue and how such changes would affect their career plans:

‘I feel a loyalty to the NHS. I believe in the NHS. I use the NHS … I want the NHS to work and I want to work for the NHS. So I don’t particularly want to go into private practice. But … I’ve been considering the different options because you need to earn money and you need to earn money in a way that works in your life.’

(F5L.F)

Many participants voiced suspicions that traditional GP practices do not fit with evolving political agendas, and linked with transfer of control over how services are delivered:

‘I get the feeling that the partnership model … the Government’s trying to like put pressure on various areas to gradually get rid of that, so that all GPs are salaried employees of the NHS, similar to hospital.’

(F5K.M)

‘There’s a looming threat of the option of partnerships being taken away and everything being run by mega corporations, or GPs then being simply salaried employees.’

(F5 FG3)

#### Workload issues

Although they enjoyed working with patients within GP practice teams, doctors’ concerns focused on escalating and unacceptable workloads, which had altered morale in practice teams:

‘They’re nice places to work, but the job is getting worse. Being a GP is getting worse. I think even in the time I’ve qualified it’s getting worse, and that’s only what, 3 years.’

(F5H.M)

‘I think it must be the workload that has increased … or maybe a combination of much more work and less staff, so that the balance has tipped, from happy GPs that have gone, and the sort of overworked GPs are left.’

(ST3G.F)

Participants described being put off by older partners who were ‘exhausted’, ‘unhappy’, ‘very burnt out’, and ‘didn’t really want to do it any more’:

*‘So I actually thought about quitting at that point, but luckily there were two younger GPs … they said, look,* [name]*, just get it done … even if it’s really awful, you can work 2 days a week and nothing is that awful for 2 days a week.’*

(ST3J.F)

Serious misgivings were linked to the responsibilities, risks, and liabilities inherent in GP partnerships with many sharing ‘horror stories’, for example, self-funded maternity leave, long notice periods, and practice closure leading to bankruptcy. Some viewed partnership as ‘a broken model’ and not an attractive option:

‘There’s a lot of uncertainty … there’s all these talks about salaried workforce, will the partnership model sustain, I don’t know. So I don’t feel like I want to put my foot into that door until it pans out.’

(F5I.F)

‘At the moment the partners I talk to, (a) they don’t seem very happy, (b) the amount of time they have to spend from a business management point of view versus the benefits that you get from being a partner, to me it doesn’t seem worth it at the moment.’

(ST3J.F)

In addition, doctors worried about delivering high-quality and safe care when overstretched, felt that increasing skill mix could alter their working patterns away from holistic care, and that a large-scale business-led service would damage continuity and personal care, and reduce professional autonomy.

#### Individual preferences

Participants appeared highly driven to choose employment compatible with their professional and lifestyle preferences. Some indicated their wish to engage and push for change within existing practice structures:

‘I definitely do want to do partnership … the only way you can change … is by being a practice partner because then you get to be the decision maker rather than just a cog in the machine.’

(ST3F.F)

‘You can’t always bring in your ideas and steer things around. You probably have to wait until somebody retires and you become the senior partner, to bring in some of your ideas.’

(F5 FG3)

Decisions could be based on first-hand experiences or reports from others, both informing how they weighed up preferred employment models; level of sessional work; portfolio career paths; leaving the NHS workforce but in many cases keeping their options open:

‘I think they do say that the work–life balance is better in Australia. You do have to work less for arguably more money and I think yeah, fair enough. I can see why that’s appealing.’

(F5C.M)

‘So many people … get so much experience doing different things within medicine and outside of medicine, and I think it’s brilliant. I think more people should be encouraged to. I think it makes you a bit more experienced and resilient later on.’

(F5I.F)

In both individual and focus group interviews, it was noted that both male and female participants reported difficulty with feeling overstretched at times and for a variety of reasons some were reluctant to work ‘full time’ as GPs:

‘I … ended up being kind of pressured into doing extra clinical session, and I think, you know, with … with hindsight, none of these things should have happened, but I think as a trainee you do feel kind of vulnerable really, because you know that you’re being assessed, and that comments that people above you make and have affected you, and the progression of your training and whether you’re going to be allowed to finish.’

(F5K.M)

‘I came back part time, it was so much easier because I had time. I had that extra 2 days a week to fit my life in and study still.’

(F5C.M)

‘When I have my kids, I may go part-time just to have time to take care of them. And maybe in that time, maybe do a Masters, ’cause I’ve always enjoyed teaching.’

(ST3G.F)

During training, several participants took sick leave as a result of work-related stress and instead of overcommitting themselves to do too much work, they would rather accept lower earnings:

*‘I don’t think I would do more than eight* [weekly sessions] *partly because I don’t think I need the money or the stress.’*

(ST3F.F)

‘People talk about getting a work–life balance, but you can’t, if you’re working more. It’s a fanciful expression, and it doesn’t ring true.’

(F5I.F)

A key difference for these emerging doctors compared with previous generations of GPs is that they have employment choices that may not previously have seemed as attractive or plentiful, including salaried contracts, regular locum work, and developing portfolio roles in academia, education, or as a ‘GP with an extended role’ in a medical specialty.

## DISCUSSION

### Summary

This article highlights aspects of GP training and preparation that new GPs perceive as being less supportive of long-term substantive GP careers. Most new GPs emerge from training with reasonable confidence in their clinical knowledge, but feel they need to consolidate this before undertaking additional roles that have not featured strongly in their training. Many are troubled by uncertainty and instability associated with traditional GP partnerships; exclusion from partnership meetings and awareness of challenging workloads and business risks add to their reticence. Among newly qualified GPs, male and female doctors prioritise work that is compatible with their desired lifestyle and are attracted to the opportunities offered by personalised or portfolio careers.

The central role of GP work in the NHS, and predictions that GP workloads will continue to rise, mean that training and retaining new GPs in substantive GP roles is increasingly important.^6^ This study is part of growing evidence that, if training experiences, transitions from training to independent practice, and partnership responsibilities are left unchanged, the traditional model of GP partnerships may be incompatible with the preparation and personal priorities of rising generations of GPs.

### Strengths and limitations

The participants’ demographic profile is broadly similar to national GP specialty training cohorts and they were geographically scattered. Doctors were included who were approaching completion of the GP specialty training programme, as well as those who had recently completed training. This facilitates gathering a more complete picture of the transitional period from in-training and post-training perspectives as participants reflected on aspirations and realities. The explorative, interview approach facilitated gathering information about aspects of the training experience that fall outside the scope of the GP curriculum, and by including both ST3 and First5 doctors the perspectives obtained are based on experiences from different stages of this transitional period.

A combination of in-depth individual interviews and discursive focus groups provided opportunities to explore individual motivation and investigate how doctors shared insights and reshaped their career plans. This study cannot gather all opinions or claim representativeness across all regions. It is not possible to report on the viewpoints of new GPs who did not participate or who may already have left the NHS or UK.

### Comparison with existing literature

This study resonates with much recent literature on patterns of recruitment to GP careers and how doctors report that their training experiences influence their career plans.^4^^,^^19^^–^21 A systematic review of GP recruitment carried out in 2017 explored some of the contextual factors,^4^ that is intrinsic preferences and organisational structures, and confirms the role of authentic experiences for career decision-making.^22^ The findings of the current study are consistent with associations identified in a regional survey of ST3 doctors, between highly-rated training and morale, and a more committed attitude to a GP career.^20^ However, in this study, rich narrative data provide a wider view of doctors’ experiences in different workplaces and links with broader perspectives^23^ than can be elucidated in survey studies.

### Implications for practice

GP training in the UK is highly rated, but this article demonstrates that there are gaps in knowledge, which hamper new GPs moving towards positions of responsibility, and there is potential for enhancing learning opportunities throughout the programme.

Pressure to satisfy CCT requirements mean that ST3 doctors often prioritise topics they perceive as important for clinical practice and formal assessments. Understanding how GP partnerships operate is less prominent in formal assessments and, in GP practices where ST3s are not encouraged to gain first-hand experience of partnership discussions, new GPs emerge with limited knowledge about decision-making and business responsibilities. Some recent initiatives, such as mentoring, buddy systems, extended GP training, Clinical Fellowship programmes, or a more structured GP career path, may help mitigate these gaps but will require future evaluation as they become more widely available.

The evidence presented here indicates a need to monitor and improve the balance between relevant learning opportunities and service provision during hospital training posts. Large-scale changes in the availability of GP specialty training programme doctors for service delivery of hospital care would, however, have major consequences for established workforce and funding arrangements.

Finally, growing opportunities for personalised, portfolio careers combined with a shift towards prioritisation of lifestyle choices may mean that the traditional model of GP partnerships may be unattractive to the next generation of GPs. This has important implications for the organisation of service delivery and primary care workforce planning.
